# Correlation between the barrier function of the intestinal epithelium and susceptibility to PEDV infection in piglets at different ages

**DOI:** 10.1186/s13567-026-01803-0

**Published:** 2026-07-08

**Authors:** Yabin Lu, Shuxian Li, Shanshan Yang, Caiying Wang, Yuguang Fu, Xin Huang, Jing Zhao, Han Shi, Jing Yang, Jianing Chen, Qingyong Guo, Ling Kuang, Guangliang Liu

**Affiliations:** 1 State Key Laboratory of Animal Disease Control and Prevention, College of Veterinary Medicine, Lanzhou University, Lanzhou Veterinary Research Institute, Chinese Academy of Agricultural Sciences, Lanzhou, 730046 China; 2 Xinjiang Key Laboratory of New Drug Study and Creation for Herbivorous Animals (XJ-KLNDSCHA), College of Veterinary Medicine, Xinjiang Agricultural University, Urumqi, 830052 China

**Keywords:** PEDV, piglets, small intestine, intestinal epithelial cells

## Abstract

Porcine epidemic diarrhoea (PED) results in vomiting, diarrhoea, dehydration, and death in pigs. To analyse the relationship between intestinal development and susceptibility to porcine epidemic diarrhoea virus (PEDV), six new-born, 1-, 2-, 3-, 4-, and 8-week-old piglets were orally inoculated with 2 × 10^7^ copies of the PEDV strain LJX01/2014 and sacrificed 48 h after infection. The results demonstrated that PEDV infection elicits diarrhoea of diverse severity across piglets of distinct ages. Moreover, viral shedding and villous atrophy were most pronounced in neonatal piglets. With increasing age, the shedding of villi gradually decreased, resulting in significantly lower villi/crypt ratios in the duodenum of newborn, 1-week-old, and 3-week-old piglets and significantly lower villi/crypt ratios in the jejunum and ileum of newborn and 1-week-old piglets than in the control. Indirect immunofluorescence and immunohistochemistry revealed a positive correlation between intraepithelial lymphocyte counts and epithelial cells and piglet age. PEDV infection led to a significant increase in intraepithelial lymphocytes and a decrease in epithelial cells, with the most severe effects observed in newborn piglets. The numbers of goblet cells, absorptive enterocytes, and enteroendocrine cells significantly decreased, whereas the numbers of Paneth cells, stem cells, and proliferating cells significantly increased with increasing piglet age. Overall, the results indicate that intestinal tissue repair increases with increasing age. Consequently, this results in a more robust intestinal epithelial barrier and reduced PEDV-induced damage to the small intestine.

## Introduction

China’s pig industry is severely affected by porcine diarrhoea caused by porcine epidemic diarrhoea virus (PEDV), which results in significant economic losses among suckling, nursery, and fattening pigs [1]. PEDV is an enveloped, single-stranded positive-sense RNA virus belonging to the *Nidovirales* order, the *Coronaviridae* family, and the *Alphacoronavirus* genus [2]. Although it was first reported in China in 1973, PEDV caused severe losses in the pig population at the end of 2010. While PEDV infects pigs of different ages, it affects mostly suckling piglets within 2 weeks of age, with morbidity and mortality rates between 80 and 100% [3].

The clinical symptoms of PEDV infection include vomiting, diarrhoea, and dehydration. Upon examination, the intestinal wall appears thin, and the intestinal lumen is filled with copious amounts of yellowish fluid [4, 5]. Histological observations reveal villous atrophy and shedding of intestinal epithelial cells. These pathological changes negatively impact piglet growth and intestinal function [6].

PEDV infection strongly affects the intestinal development of piglets. As a vital component of the digestive system, the intestine not only plays a crucial role in food digestion and absorption but also serves as the primary barrier against pathogenic microorganism invasion [7]. Healthy intestinal development is essential to ensure the health of pigs. As the primary site for nutrient absorption, the small intestine undergoes continuous, stage-specific adaptive changes in villus length, width, crypt depth, and morphology to sustain its absorptive function. The distribution and quantity of intestinal epithelial cells in the intestine are key factors influencing intestinal function and the development of pigs [8]. Intestinal epithelial cells and their secretions are the first barrier for intestinal tissue to come into contact with the outside world. The intestinal mucosal immune system, which is composed of intestinal epithelial cells, secretory cells, and intraepithelial lymphocytes, is critical for host defence [9, 10].

PEDV infection-induced damage in pigs is closely related to intestinal development. This study investigated the developmental and morphological alterations in various intestinal tissues among piglets at different ages. The results highlight intestinal development, which includes the villus architecture, epithelial cell composition, and presence of IELs, as a primary defence against PEDV pathogenesis. These findings provide a scientific foundation for the development of age-targeted interventions and inform research on mucosal immunity against enteric coronaviruses.

## Materials and methods

### Viral material

The virulent PEDV field strain LJX01/2014 was obtained from diarrheal piglets from a pig farm in LiuJiaXia, Gansu, China. In brief, the small intestinal contents of piglets with diarrhoea were resuspended in sterile PBS (1:1) and centrifuged at 12 000 rpm for 20 min at 4 °C to remove intestinal bacteria. The supernatant was subsequently collected, subjected to filtration through 0.45 μm filters, and quantified for PEDV loads via probe-based real-time RT‒qPCR. TGEV, PDCoV, and PEAV were not detectable by the multiplex fluorescence quantitative PCR method established in the laboratory. The PEDV stock was preserved at -80 °C until use.

### Animals and animal experiments

All experimental procedures and animal care protocols were approved by the Guidelines for the Care and Use of Laboratory Animals of Lanzhou Veterinary Research Institute (LVRI), Chinese Academy of Agricultural Sciences, China. To obtain PEDV-free piglets, piglets of different ages and sows were screened by real-time RT‒qPCR and ELISA, which are specific for both the PEDV genome and antibodies. After screening, forty PEDV-free Duroc-Landrace-Yorkshire piglets (eight piglets for each group of new-born and 1-week-old piglets, six pigs for each group of 2-week-old, 3-week-old, 4-week-old, and 8-week-old piglets) were purchased from a pig farm located in Dingxi, Gansu Province, and housed in isolated animal rooms. The piglets in each experimental group were from the same sow. All the pigs used in this experiment were from the same farm and same commercial breed. The newborn piglets, 1-week-old piglets, and 2-week-old piglets were fed a milk substitute (Anyou, China), while the 3-, 4-, and 8-week-old piglets were fed regular feed.

Piglets from each age group were randomly divided into a challenge group and a mock control group. For the six challenge groups, the piglets were orally inoculated with 2 × 10^7^ copies of the PEDV LJX01/2014 strain. Mock-infected piglets were orally inoculated with an equal volume of sterile PBS. The rectal temperature of each piglet was measured every 12 h after inoculation, while the rectal swabs of each piglet were collected every 12 h for detection of viral shedding via real-time RT‒qPCR (the TaqMan probe used for detecting PEDV is presented in Table 2). In addition, faecal samples were collected directly from the rectum of each piglet and scored blindly and independently by two investigators to evaluate the severity of diarrhoea (0, normal faeces; 1, mixed stool samples containing both solid and pasty feces; 2, pasty faeces; 3, semiliquid faeces; and 4, liquid faeces). At 48 h post-infection (hpi), all the piglets were sacrificed and necropsied. Sections from the duodenum, jejunum, and ileum were harvested for viral load detection and evaluation of pathological changes.

### Morphological analysis

The duodenum, jejunum, and ileum tissues were fixed in paraformaldehyde for 48 h, dehydrated through a graded series of ethanol concentrations (75%, 85%, 95%, 100%) for 1 min each, and embedded in paraffin wax. Longitudinal Sects. (5 µm thick) were cut using a tissue slicer and dried. The paraffin sections were deparaffinized in xylene for 20 min and then in alcohol (100%, 95%, 85%, 75%) for 10 min each. They were rinsed under running water for 20 min, stained with eosin for 2 min, differentiated in hydrochloric acid for 5 s, stained with haematoxylin for 6 min, dehydrated in alcohol (85%, 95%, 100%) for 10 min each, and observed after being mounted on slides.

### Immunofluorescence

The frozen tissue sections from the duodenum, jejunum, and ileum were incubated in 4% paraformaldehyde for 20 min, followed by glycine fixation for 5 min. The samples were subsequently permeabilized with 1% Triton X-100 for 20 min, washed with PBS for 5 min, and incubated overnight with the primary antibody. After the samples were washed with PBS at room temperature, they were incubated with an HRP-conjugated goat anti-mouse (1:500) secondary antibody for 2 h. After 5 min of DAPI staining, the samples were mounted on slides and observed under a confocal laser-scanning microscope (Zeiss). The antibodies used in this study are listed in Table 1.

**Table 1:** **Information about the antibodies used for immunohistochemistry and immunofluorescence**

Antibody	Dilution rate	Size	Company
PEDV-N	1:200	\	Self-made
KI67	1:600	250 µg/mL	BD Biosciences
Lgr5	1:100	100 µL	Merck
Lysozyme	1:100	500 µL	Invitrogen
Villin	1:200	200 µg/mL	SantaCruz
Chr-A	1:200	200 µg/mL	BD Biosciences
CGA	1:100	100 µL	Merck
SOX9	1:100	100 µL	CST
SABC-POD(F) (mouse IgG)	\	1 kit	Boster
SABC-POD(F) (rabbit IgG)	\	1 kit	Boster

### Immunohistochemistry

The tissue sections were deparaffinized and dehydrated, after which the antigens were recovered from the tissue sections in citrate buffer (pH 6) at 90 ~ 95 °C for 15 min and then incubated with 0.3% hydrogen peroxide for 15 min. The sections were rinsed three times with distilled water, blocked with 5% skim milk at 37 °C for 40 min, and incubated overnight with antibodies. After being rinsed with PBS, the sections were incubated with an HRP-conjugated goat anti-mouse (1:500) secondary antibody at 37 °C for 40 min. SABC (streptavidin–biotin complex; Bioss Beijing) was added, and the samples were incubated at 37 °C for 30 min. After the sections were rinsed with PBS, DAB (diaminobenzidine) staining was performed until a brown precipitate formed. The samples were subsequently stained with haematoxylin for 5 min, dehydrated, mounted in neutral resin, and observed under an optical microscope. The antibodies used in this study are listed in Table 1.

### PAS staining

The paraffin-embedded sections were deparaffinized, dehydrated and oxidized with periodic acid for 12 min. After they were rinsed with distilled water, the sections were incubated in Schiff’s reagent in a darkened oven at 37 °C for 30 min and rinsed under running water for 10 min. Mayer’s haematoxylin staining was performed for 30 s, after which they were mounted. Six random microscopic fields were selected from each tissue section, and the number of goblet cells in the villous epithelium units was quantified via Image-Pro Plus.

### RNA extraction and real-time quantitative PCR

The collected duodenum, jejunum, and ileum tissue was placed into 2 mL centrifuge tubes and rapidly frozen in liquid nitrogen. The tissues were subsequently ground and crushed, and 1 mL of TRIzol was subsequently added to fully lyse the crushed tissue. Afterward, 200 µL of chloroform was added, thoroughly mixed, left to stand at room temperature for 10 min, and centrifuged at 4 °C for 10 min at 12 000 rpm. The supernatant was carefully collected into DNase-free tubes, and an equal amount of isopropanol was added. After thorough mixing, the mixture was left overnight at -20 °C and then centrifuged at 4 °C for 10 min at 12 000 rpm. The supernatant was discarded, and 1 mL of 75% ice-cold ethanol was added, followed by centrifugation at 4 °C for 10 min at 12 000 rpm. The supernatant was discarded, and 30 µL of RNase-free water was added and thoroughly mixed. Finally, the RNA content was measured by a NanoDrop spectrophotometer and stored for later use.

For reverse transcription, 4 µL of gDNA wiper mix was mixed with 1 µg of template RNA in a total volume of 16 µL. The mixture was thoroughly mixed and incubated at 42 °C for 2 min, after which 5 × HiScriptⅡ qRT SuperMix Ⅱ was added. The reverse transcription reaction was carried out at 50 °C for 15 min, followed by 85 °C for 5 s to complete the reverse transcription. The resulting cDNA samples were stored at -20 °C for future use.

To determine the relative expression levels of cellular markers, a 1 µL cDNA sample was subjected to the following conditions: predenaturation at 95 °C for 5 min, denaturation at 95 °C for 10 s, annealing at 60 °C for 20 s, and extension at 72 °C for 20 s, with a total of 40 cycles of C1000 Touch™ Thermal Cycler (Bio-Rad). The primers used for detecting cellular markers are presented in Table 2.

**Table 2:** **Oligonucleotide PCR primers**

Gene	Orientation	Primer Sequence (5 → 3)
PEDV-M	Forward	GAT ACT TTG GCC TCT TGT GT
	Reverse	CAC AAC CGAATG CTA TTG ACG
Taqman probe	\	AGC ATC CTT ATG GCT TGC ATC
GAPDH	Forward	GATGGGCGTGAACCATGAGA
	Reverse	CATGGACCGTGGTCATGAGT
KI67	Forward	GGAGGCAATATTACATAATTTCA
	Reverse	CAGGGTCAGAAGAGAAGCTA
Mucin2	Forward	GGCTGCTCATTGAGAGGAGT
	Reverse	ATGTTCCCGAACTCCAAGG
Lgr5	Forward	GAGCCTGGGAAAGCAAACC
	Reverse	GGACAAATGCCACGGAAGA
Villin	Forward	TTGTAGCGGAGATGAGCGGGAGA
	Reverse	CGGGGAGTGATGACCAGGGTTTC
Chr-A	Forward	GACCTCGCTCTCCAAGGAGCCA
	Reverse	TGTGCGCCTGGGCGTTTCTT
Claudin-3	Forward	AAGCCAAGATCCTCTACTCC
	Reverse	GTAGTCCTTGCGGTCGTA
E-cadherin	Forward	AAATGCTTAGCTGGTGGGGAC
	Reverse	GCCTCCCATTGCTAACACCT
Occludin	Forward	ATCAACAAAGGCAACTCT
	Reverse	GCAGCAGCCAT0GTACTCT
Zo-1	Forward	AGCCCGAGGGGTGTTT
	Reverse	GGTGGGAGGATGCTGTTG

### Statistical analysis

All the data were analysed by SPSS. Significant differences were determined via one-way analysis of variance (*P ≤ 0.05; ** *P* ≤ 0.01; *** *P* ≤ 0.001; **** *P* ≤ 0.0001). The data are presented as the means ± SEMs (standard error of the mean). All the graphs were generated via GraphPad Prism software 7.0.

## Results

### Pathogenicity of PEDV in piglets of different ages

**Figure 1:**
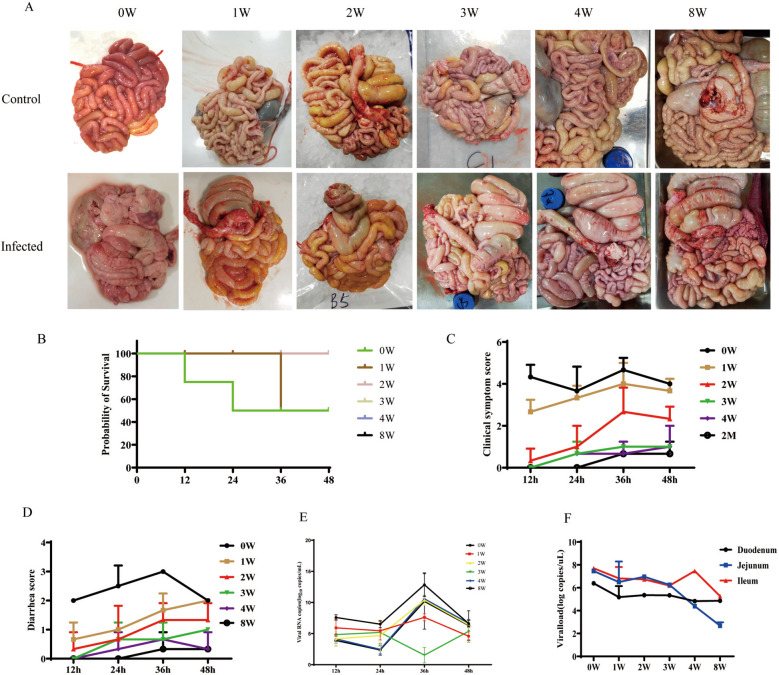
**Clinical symptoms of PEDV infection**. **A** Clinical symptoms in the intestines of pigs of all ages were observed. **B** Survival curve. **C** Clinical symptoms. **D** Diarrheal score. **E** Viral loads of the anal swabs. **F** Viral loads in the intestines.

The impact of PEDV infection varies among piglets of different ages. Neonatal piglets display the earliest and most severe symptoms, characterized by excessive watery diarrhoea with yellowish liquid faeces. One-week-old piglets showed a transition from loose stools to yellowish liquid faeces over time. The other age groups exhibited varying degrees of diarrhoea, mostly presenting as loose or soft stools without significant signs of depression or loss of appetite. Following pathological examination, both newborn piglets and 1-week-old piglets presented typical lesions of porcine epidemic diarrhoea characterized by a small intestine filled with large amounts of yellowish liquid and thin and transparent intestinal walls. In contrast, while the intestinal lumen did not significantly change in the other age groups, and there were no signs of bleeding (Figure 1A). Survival curves, clinical symptoms, and diarrhoea patterns revealed that the mortality rate was highest in newborn and 1-week-old piglets, with no deaths occurring in the pigs older than 2 weeks (Figure 1B-D). The newborn piglets were the most severely affected, followed by the 1-week-old piglets, while the impact on the other age groups was less pronounced. The viral load in rectal swabs revealed that all piglets of different ages were infected with PEDV, with the viral load gradually increasing with infection time, peaking at 36 h (Figure 1E). The viral load results for the duodenum, jejunum, and ileum of pigs of different ages revealed that the viral load was highest in the ileum of newborn piglets. As the age of the infected piglets increased, the viral load in the intestinal tissues of different age groups gradually decreased (Figure 1F).

### Morphological and histological analysis of the small intestine and PEDV antigen distribution in the intestine

**Figure 2:**
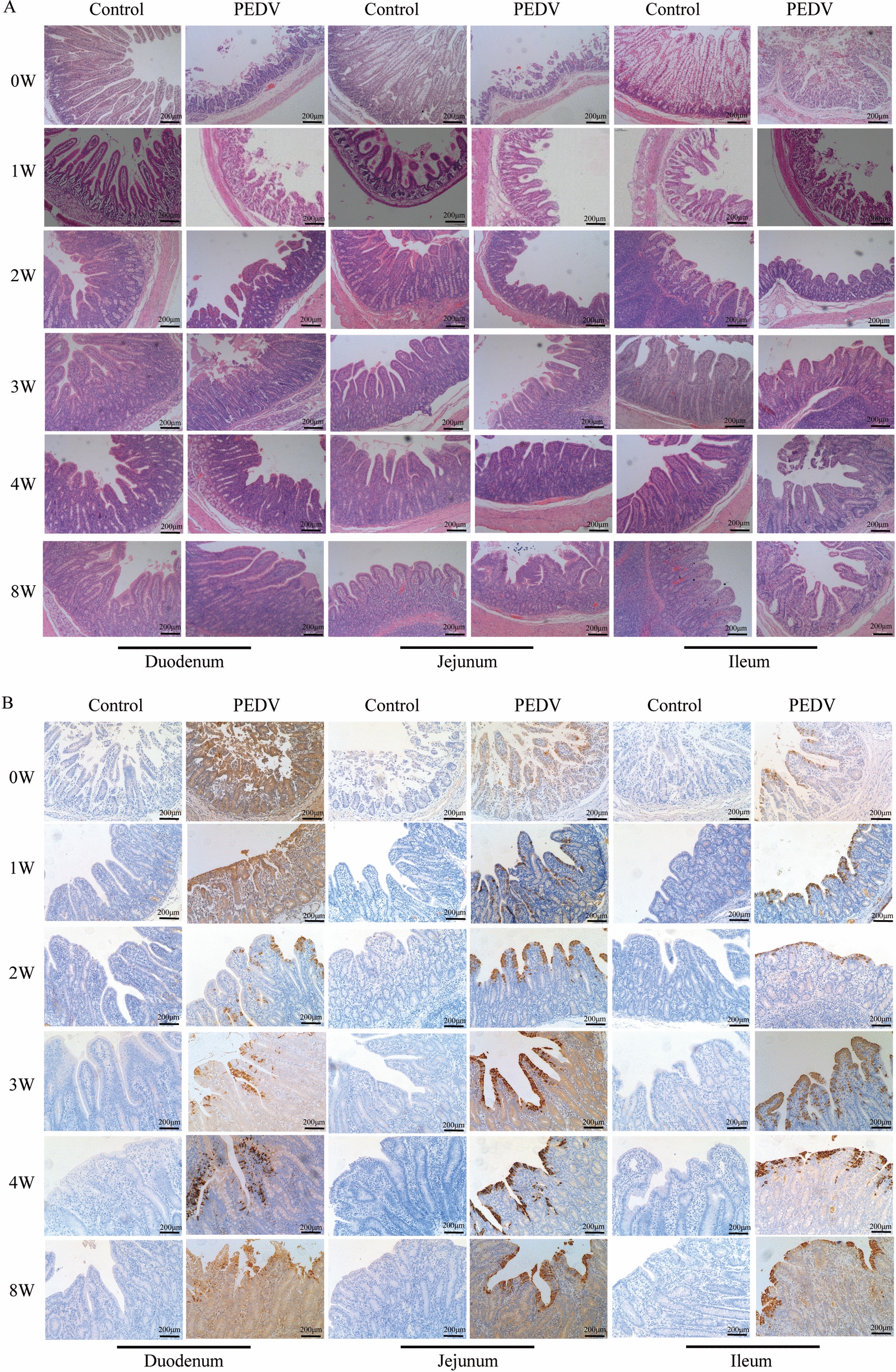
**Pathogenicity induced by PEDV infection.** **A** H&E staining of different segments of the small intestine from different age groups. **B** Villus: crypt ratio. **C** Number of intestinal intraepithelial lymphocytes. **D** Immunohistochemical analysis for detection of the PEDV N protein. In all the graphs, the data are shown as the mean ± SEM. The results are presented as follows: **, *P* < 0.01; ****, *P* < 0.0001.

The effects of PEDV infection on the morphology of the small intestine were analysed. PEDV-infected piglets of different ages all exhibited villous atrophy in the small intestine, with the most severe effects observed in the newborn and 1-week groups. In newborn piglets, the villi of the jejunum and ileum were atrophied, intestinal epithelial cells were shed, the lamina propria was exposed, and severe damage was evident. In 1-week-old piglets, the duodenum and ileum were significantly damaged and characterized by noticeable villus atrophy and shedding of intestinal epithelial cells. The jejunum exhibited incomplete villus morphology, which was characterized by the shedding of epithelial cells at the tip of the villi. Although villous atrophy was observed in the duodenum, jejunum, and ileum of 2-week-old piglets, no significant atrophy was observed. In 3-week-old piglets, obvious villous atrophy was observed in the duodenum, whereas villi atrophy was observed in the jejunum and ileum. No apparent villi atrophy in the duodenum, jejunum, or ileum was observed in the 4-week-old or 8-week-old piglets following PEDV infection (Figure 2A).

Next, the intestinal villus/crypt ratio before and after PEDV infection was statistically analysed in piglets of different ages. Compared with control piglets, PEDV-infected piglets presented significantly different villi/crypt ratios in different intestinal tissues. Additionally, the changes in the villus/crypt ratio induced by PEDV infection decreased with increasing piglet age. Compared with that of the control piglets, a significantly lower duodenal villi/crypt ratio (*P* < 0.01) was observed in the newborn, 1-week-old, and 3-week-old piglets; a significantly lower jejunum villi/crypt ratio (*P* < 0.01) was observed in the newborn and 1-week-old piglets, and a significantly lower ileal villi/crypt ratio (*P* < 0.0001) was observed in the newborn and 1-week-old piglets. The villi/crypt ratio did not significantly differ between the other groups and the control group (Figure 2B).

The number of intestinal intraepithelial lymphocytes in different intestinal tissues from piglets of different ages was subsequently determined. Additionally, significant changes in the number of intestinal intraepithelial lymphocytes were observed in different intestinal tissues of piglets at different ages before and after PEDV infection. Compared with that in the control piglets, the number of intestinal intraepithelial lymphocytes in the duodenum was significantly greater in the 2-, 3-, and 4-week-old piglets (*P* < 0.0001) but significantly lower in the eight-week-old piglets (*P* < 0.0001), whereas no significant difference between the control and the other groups was detected (Figure 2C).

The results of immunohistochemical staining for the PEDV N protein revealed that the viral antigen was present on the villi of the duodenum, jejunum, and ileum in challenged piglets and that compared with mock-infected piglets, challenged piglets presented obvious brown precipitation reactions (Figure 2D).

### Effects of PEDV infection on the expression of tight junction proteins in the intestines of piglets of different ages

**Figure 3:**
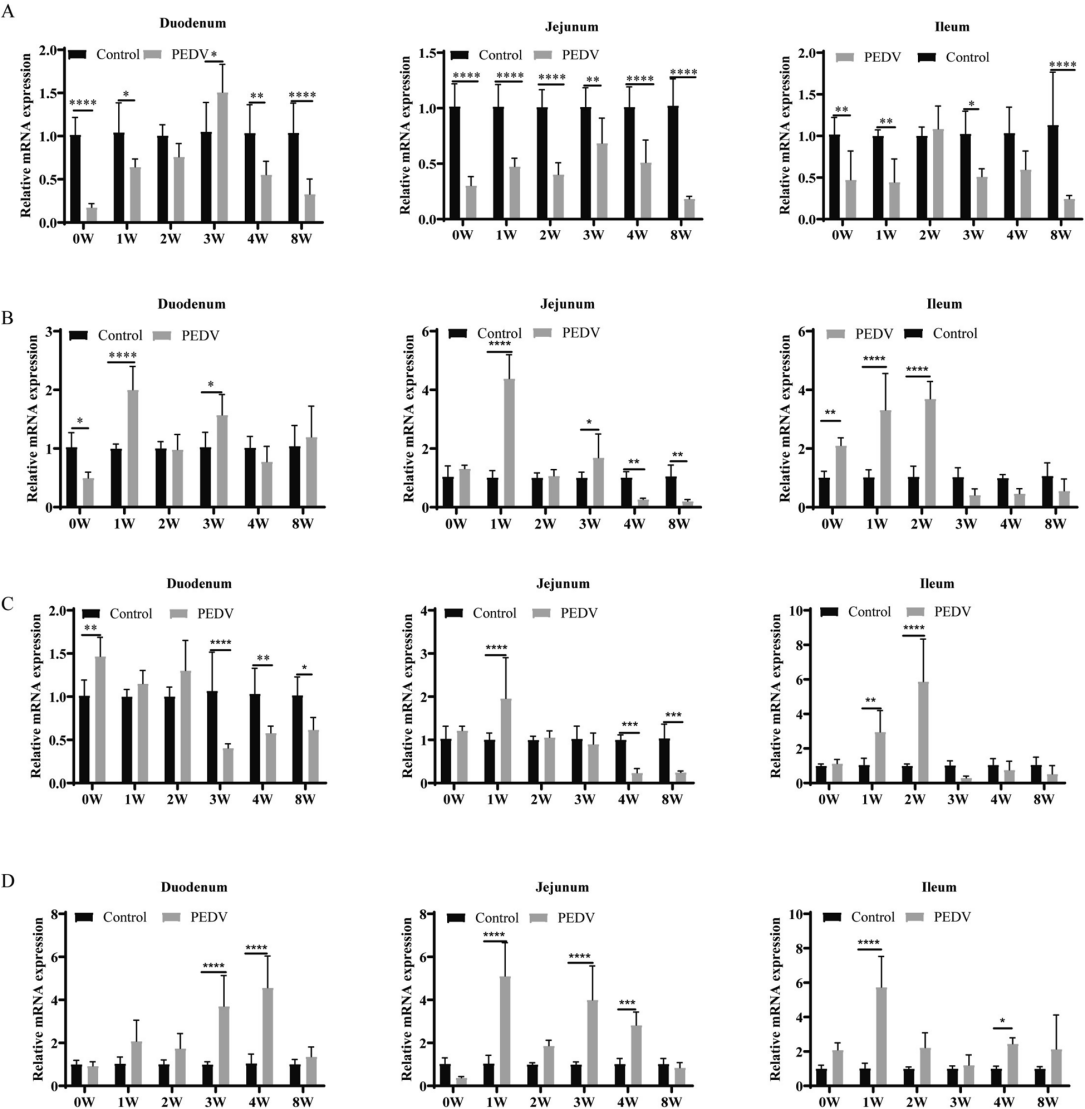
**Real-time PCR was used to measure the mRNA expression levels of ZO-1, occludin, claudin-3, and E-cadherin in the duodenum, jejunum, and ileum**. **A** mRNA expression level of ZO-1. **B** mRNA expression level of occludin. **C** mRNA expression levels of claudin-3. **D** mRNA expression level of E-cadherin. In all the graphs, the data are shown as the mean ± SEM. The results are presented as follows: *, *P* < 0.05; **, *P* < 0.01; ***, *P* < 0.001; and ****, *P* < 0.0001.

The effects of PEDV infection on the mRNA expression levels of ZO-1, occludin, claudin-3, and E-cadherin in the intestinal mucosa of piglets of different ages are shown in Figure 3. PEDV infection resulted in decreased ZO-1 expression levels in different intestinal tissues of piglets of different ages. Compared with those in control piglets, ZO-1 expression levels were significantly lower in the duodenum of PEDV-infected newborn piglets and 1-, 4-, and 8-week-old piglets (*P* < 0.05) but significantly greater in PEDV-infected 3-week-old piglets (*P* < 0.05). Additionally, compared with those in the control piglets, ZO-1 expression levels were significantly lower in the jejunum of PEDV-infected piglets in all groups (*P* < 0.01) and significantly lower in the ileum of PEDV-infected newborn piglets and 1-, 3-, and 8-week-old piglets (*P* < 0.05) (Figure 3A).

PEDV infection significantly altered occludin levels in different intestinal tissues of piglets. Compared with those in the control piglets, occludin expression levels were significantly lower in the duodenums of the piglets infected with PEDV (*P* < 0.05) and significantly greater in the duodenums of the piglets infected with PEDV at 1 week and 3 weeks (*P* < 0.05). Additionally, compared with those in the control piglets, occludin expression levels were significantly greater in the jejunum of PEDV-infected 1-week-old and 3-week-old piglets (*P* < 0.05), significantly lower in the jejunum of PEDV-infected 4-week-old and 8-week-old piglets (*P* < 0.01), and significantly greater in the ileum of PEDV-infected newborn, 1-week-old and 2-week-old piglets (*P* < 0.05) (Figure 3B).

PEDV infection resulted in significantly different claudin-3 gene expression patterns in different intestinal tissues of piglets. Compared with those in the control piglets, claudin-3 expression levels were significantly greater in the duodenum of newborn piglets infected with PEDV (*P* < 0.01) and significantly lower in the duodenum of piglets infected with PEDV at 3, 4, and 8 weeks (*P* < 0.05). Additionally, compared with those in the control piglets, claudin-3 expression levels were significantly greater in the jejunum of PEDV-infected 1-week-old piglets (*P* < 0.05) and significantly lower in the jejunum of PEDV-infected 4-week-old and 8-week-old piglets (*P* < 0.01). Finally, compared with those in the control piglets, claudin-3 expression levels were significantly greater in the ileum of PEDV-infected 1-week-old and 2-week-old piglets (*P* < 0.01), whereas no significant difference between the other groups was observed (Figure 3C).

PEDV infection resulted in significantly increased E-cadherin expression levels in different intestinal tissues of piglets of different ages. Compared with those in the control piglets, E-cadherin expression levels were significantly greater in the duodenum of PEDV-infected 3-week- and 4-week-old piglets (*P* < 0.01), significantly greater in the jejunum of PEDV-infected 1-, 3-, and 4-week-old piglets (*P* < 0.01), and significantly greater in the ileum of PEDV-infected 1- and 4-week-old piglets (*P* < 0.05). No significant difference between the other groups was observed (Figure 3D).

### Changes in intestinal epithelial absorption cells in the small intestine of piglets of different ages

**Figure 4:**
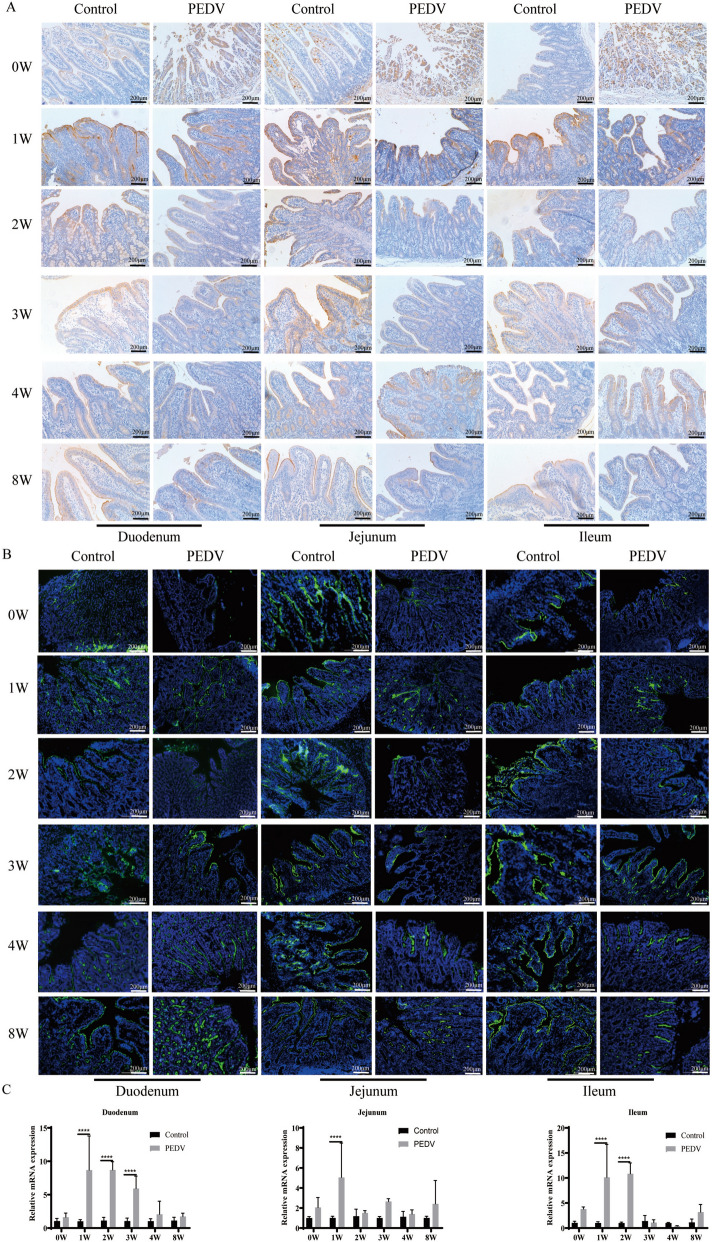
**Expression of enterocytes in the small intestine.** **A** Immunohistochemical staining of enterocytes. **B** Immunofluorescence staining of enterocytes. **C** The mRNA expression level of Villin. In all the graphs, the data are shown as the mean ± SEM. The results are designated as follows: ****, *P* < 0.0001.

Immunohistochemistry and immunofluorescence revealed that intestinal epithelial absorptive cells were widely distributed in the intestinal villi and crypts, with obvious specific staining in the peripheral villi of different intestinal tissues of piglets at different ages. Compared with that in the control piglets, specific staining was absent at the top of the intestinal villi in the PEDV-infected newborn, 1-week-old, and 2-week-old piglets (Figure 4A and B).

Next, RT‒qPCR analysis revealed significant differences in the expression levels of villin mRNA, a marker of intestinal epithelial cells, in different intestinal tissues of PEDV-infected piglets. Compared with those in the control piglets, villin expression levels were significantly greater in the duodenum of PEDV-infected 1-, 2-, and 3-week-old piglets (*P* < 0.01), significantly greater in the jejunum of 1-week-old piglets (*P* < 0.0001), and significantly greater in the ileum of 1- and 2-week-old piglets (*P* < 0.0001) (Figure 4C).

### Changes in the number of goblet cells in the small intestine of piglets of different ages

**Figure 5:**
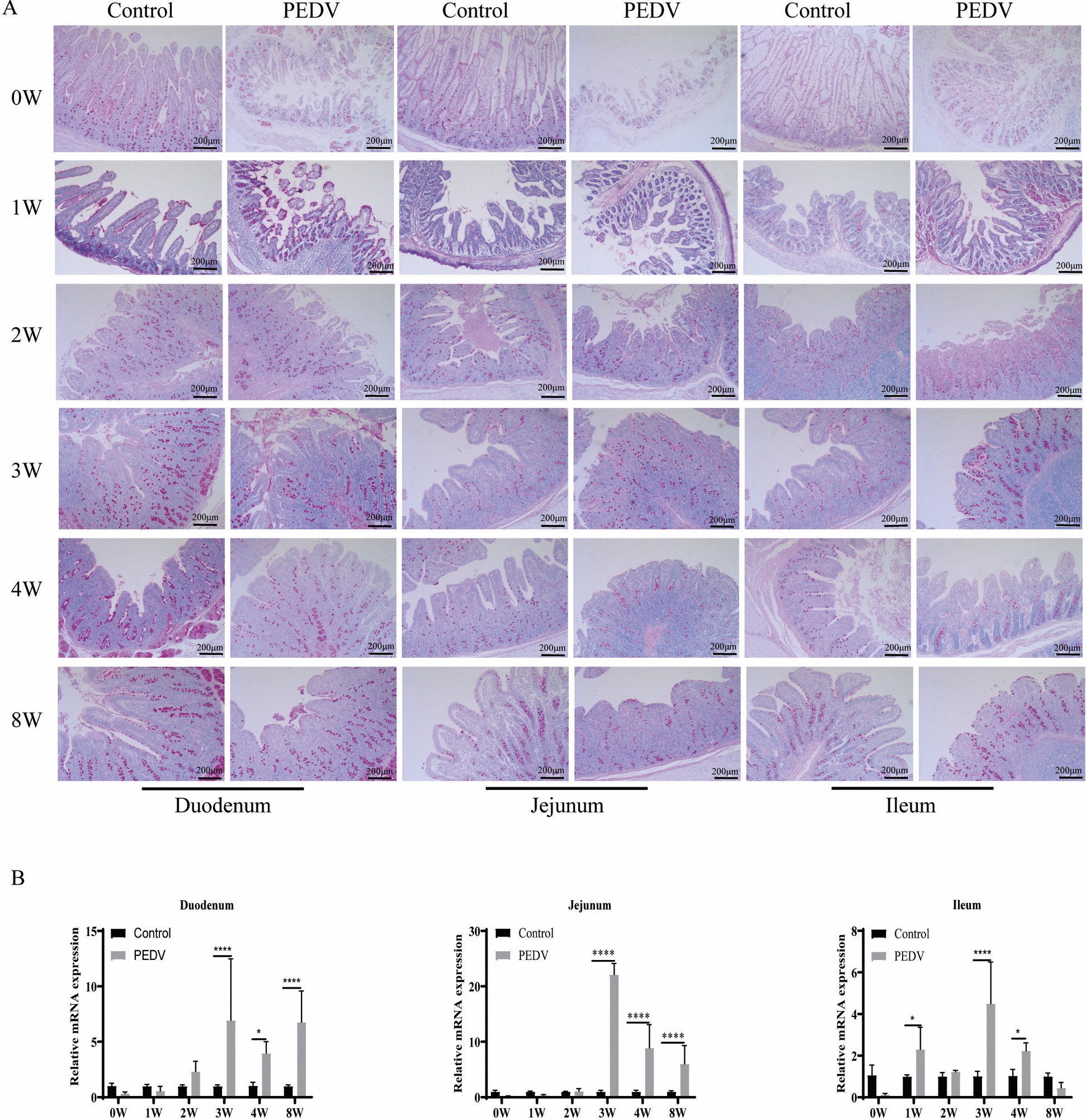
**Expression of goblet cells in the small intestine.** **A** Immunohistochemical staining of goblet cells. **B** The number of goblet cells in the small intestine. **C** mRNA expression level of Mucin2. In all the graphs, the data are shown as the mean ± SEM. The results are presented as follows: *, *P* < 0.05; ****, *P* < 0.0001.

PAS staining, which is used to label goblet cells, revealed that the goblet cells appeared primarily purple‒red in colour and were distributed mainly between the mucosal epithelium and intestinal glands (Figure 5A). In control piglets, the number of goblet cells increased with age. Compared with that of the control piglets, the number of goblet cells was significantly lower in the duodenum of the PEDV-infected newborn piglets at 1, 2, and 3 weeks (*P* < 0.0001), significantly lower in the jejunum of the newborn piglets at 1, 2, 3, 4, and 8 weeks (*P* < 0.05), and significantly lower in the ileum of the newborn piglets at 1, 2, and 3 weeks (*P* < 0.0001), while significant differences between the other groups were lacking. These observations indicated that PEDV infection resulted in decreased goblet cell numbers in different intestinal tissues of piglets of different ages. Notably, the number of goblet cells gradually stabilized as the age of the PEDV-infected piglets increased (Figure 5B).

RT‒qPCR analysis of the expression of the goblet cell marker mucin2 before and after infection in different intestinal tissues of piglets at different ages revealed significantly higher expression levels in the duodenum of PEDV-infected 3-, 4-, and 8-week-old piglets than in that of control piglets (*P* < 0.05). Compared with that in the control piglets, mucin expression was significantly greater in the jejunum of PEDV-infected 4-week-old and 8-week-old piglets (*P* < 0.0001) and in the duodenum of 1-, 3-, 4-, and 8-week-old piglets (*P* < 0.05), while no significant differences were observed between the other groups (Figure 5C).

### Changes in Paneth cells in the small intestine of piglets of different ages

**Figure 6:**
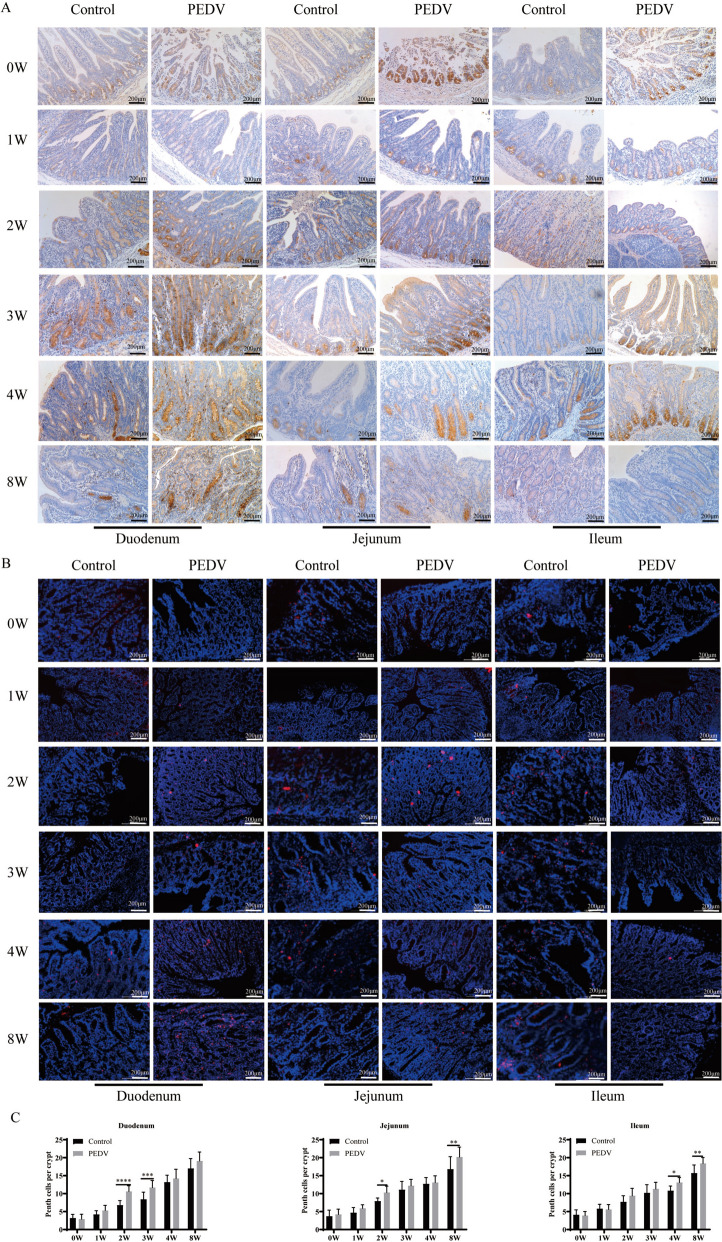
**Expression of Paneth cells in the small intestine.** **A** Immunohistochemical staining of Paneth cells. **B** Immunofluorescence staining of Paneth cells. **C** The number of Paneth cells in the small intestine. In all the graphs, the data are shown as the mean ± SEM. The results are presented as follows: *, *P* < 0.05; **, *P* < 0.01; ***, *P* < 0.001; and ****, *P* < 0.0001.

Immunohistochemistry and immunofluorescence indicated that Paneth cells were located mainly in the intestinal crypts of different intestinal tissues of piglets of different ages (Figure 6A and B).

In control piglets, the number of Paneth cells increased with increasing piglet age. Following PEDV infection, the Paneth cell number changed significantly in different intestinal tissues of piglets at different ages. In fact, compared with that in the control piglets, the number of Paneth cells was significantly greater in the duodenum of 2-week-old and 3-week-old piglets (*P* < 0.001), in the jejunum of PEDV-infected 2-week-old and 8-week-old piglets (*P* < 0.05), and in the ileum of PEDV-infected 4-week-old and 8-week-old piglets (*P* < 0.05) (Figure 6C).

### Changes in the number of proliferating cells in the small intestine of piglets of different ages

**Figure 7:**
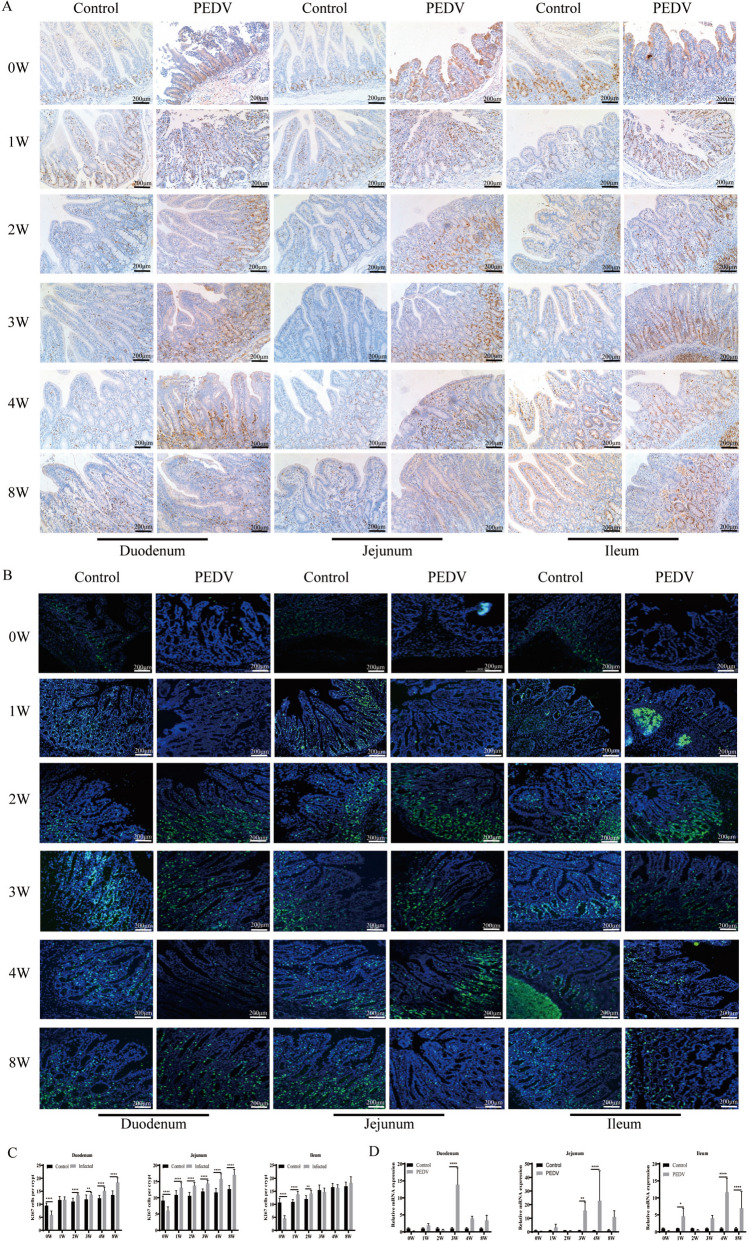
**Expression of proliferating cells in the small intestine.** **A** Immunohistochemical staining of proliferating cells. **B** Immunofluorescence staining of proliferating cells. **C** The number of proliferating cells in the small intestine. **D** The mRNA expression level of Ki67. In all the graphs, the data are shown as the mean ± SEM. The results are presented as follows: *, *P* < 0.05; **, *P* < 0.01; ***, *P* < 0.001.

Immunohistochemistry and immunofluorescence revealed that proliferating cells were widely distributed in the intestinal villi and crypts and increased with increasing piglet age (Figure 7A and B). In PEDV-infected piglets, the number of proliferating cells differed significantly. Compared with that in the control piglets, the number of proliferating cells in the duodenum was significantly lower in the PEDV-infected newborn piglets (*P* < 0.001) but significantly greater in the 2-, 3-, 4-, and 8-week-old piglets (*P* < 0.01). Compared with that of the control piglets, the number of proliferating cells was significantly lower in the jejunum of newborn piglets (*P* < 0.001) but significantly greater in 1-, 2-, 3-, 4-, and 8-week-old piglets (*P* < 0.01). Compared with that in the control piglets, the number of proliferating cells in the ileum was significantly lower in newborn piglets (*P* < 0.001) but significantly greater in 1-week-old and 2-week-old piglets (*P* < 0.01), while no significant difference between the other groups was observed (Figure 7C).

RT‒qPCR analysis revealed that, compared with those in the control piglets, the expression levels of the proliferating cell marker KI-67 were significantly greater in the duodenum of 3-week-old piglets (*P* < 0.0001), significantly greater in the jejunum of 3-week-old piglets and 4-week-old piglets (*P* < 0.01), and significantly greater in the ileum of 1-, 4-, and 8-week-old piglets (*P* < 0.0001); however, there were no significant differences between the other groups (Figure 7D).

### Changes in intestinal enteroendocrine cells in the small intestine of piglets of different ages

**Figure 8:**
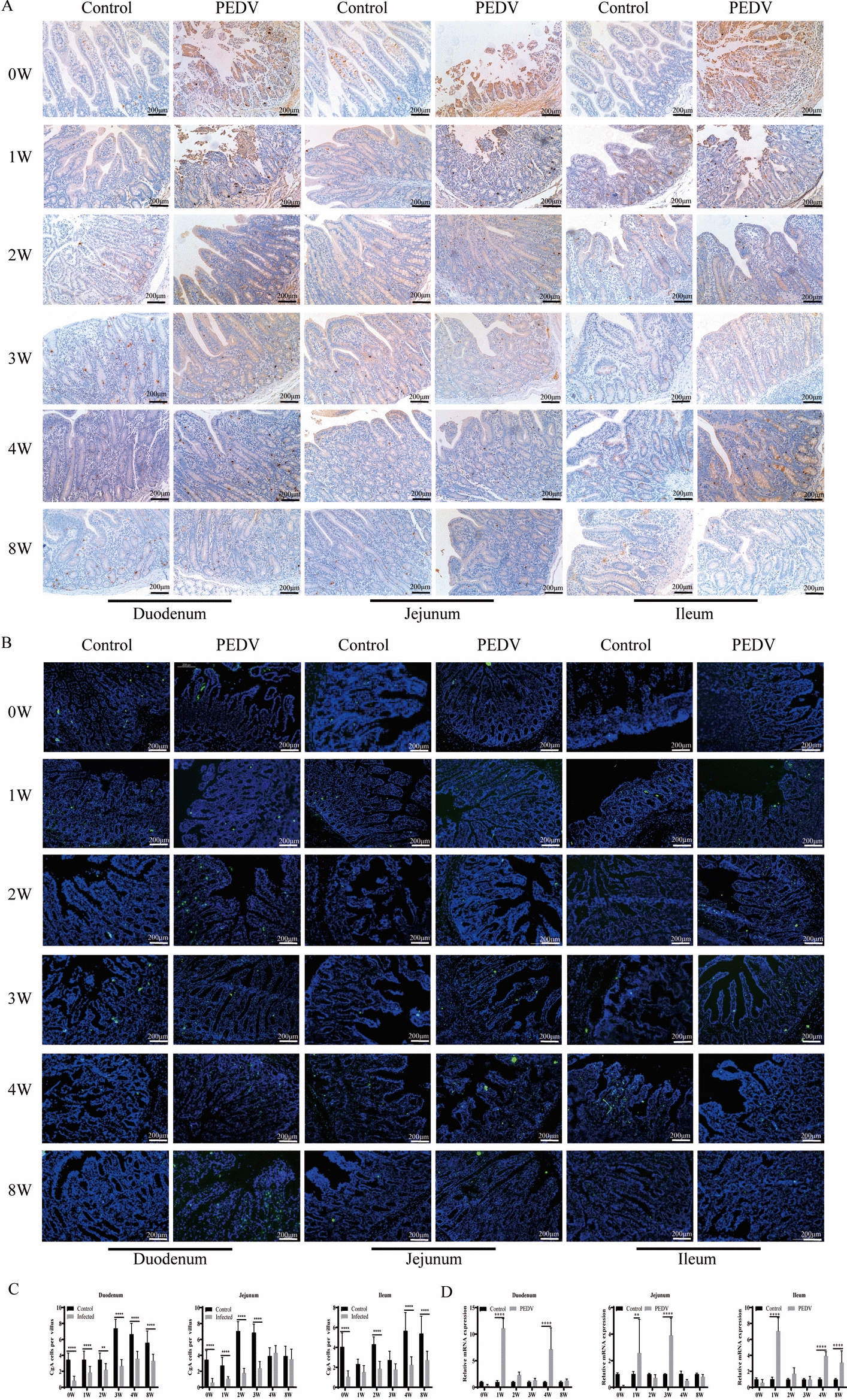
**Expression of secretory cells in the small intestine.** **A** Immunohistochemical staining of secretory cells. **B** Immunofluorescence staining of secretory cells. **C** The number of secretory cells in the small intestine. **D** The mRNA expression level of Chr-A. In all the graphs, the data are shown as the “mean ± SEM”. The results are presented as follows: *, *P* < 0.05; **, *P* < 0.01; ***, *P* < 0.001; and ****, *P* < 0.0001.

Immunohistochemistry and immunofluorescence revealed a wide distribution of enteroendocrine cells in the intestinal villi and crypts as well as increasing cell numbers with increasing age (Figure 8A and B).

Compared with those of the control piglets, the numbers of intestinal endocrine cells were significantly lower in the duodenums of PEDV-infected newborns; 1-, 2-, 3-, 4-, and 8-week-old piglets (*P* < 0.01); in the jejuna of PEDV-infected newborns; 1-, 2-, and 3-week-old piglets (*P* < 0.0001); and in the ilea of PEDV-infected newborns; and 2-, 4-, and 8-week-old piglets (*P* < 0.0001). No significant differences between the other groups were apparent (Figure 8C).

RT‒qPCR analysis revealed that compared with those in the control piglets, the expression levels of Chr-A, an enteroendocrine cell marker, were significantly greater in the duodenal endocrine cells of PEDV-infected 1-, 4-, and 8-week-old piglets (*P* < 0.0001), significantly greater in the jejunum of PEDV-infected 1- and 3-week-old piglets (*P* < 0.01), and significantly greater in the ileum of 1-, 4-, and 8-week-old piglets (*P* < 0.0001). No significant differences were observed between the other groups (Figure 8D).

### Changes in stem cells in the small intestine of piglets of different ages

**Figure 9:**
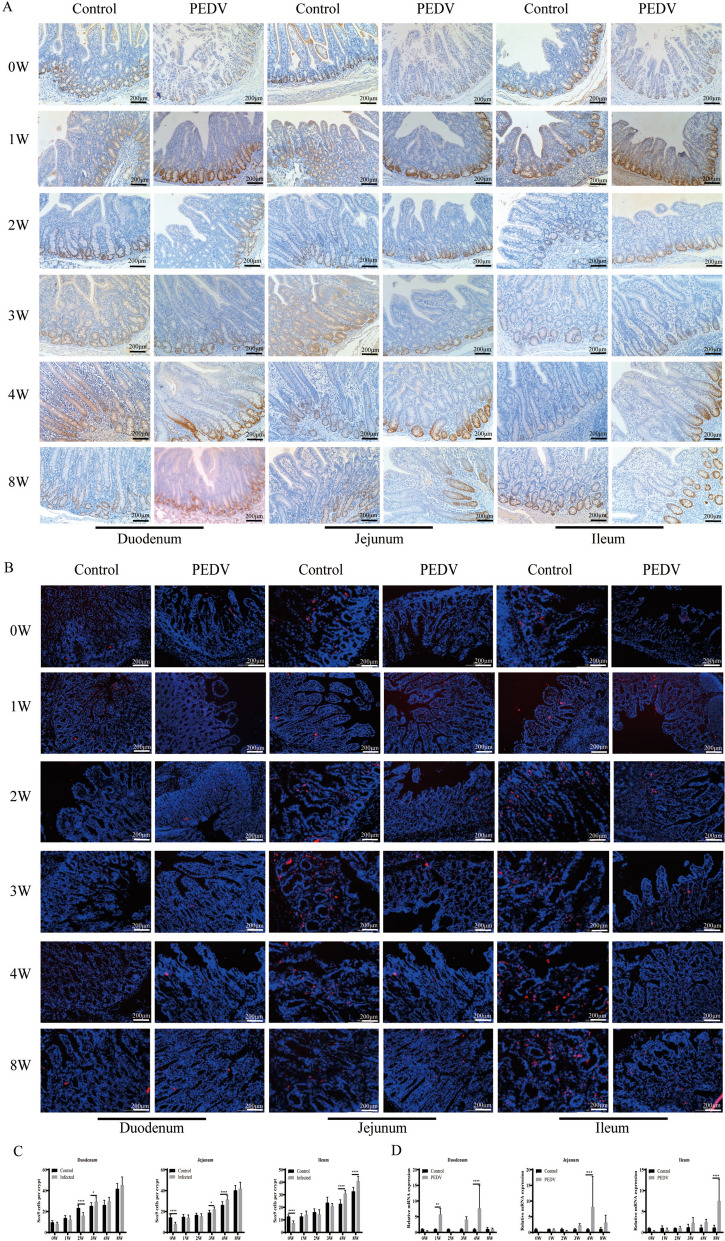
**Stem cell expression in the small intestine.** **A** Immunohistochemical staining of stem cells. **B** Immunofluorescence staining of stem cells. **C** The number of stem cells in the small intestine. **D** The mRNA expression level of Lgr5. In all the graphs, the data are shown as the mean ± SEM. The results are presented as follows: *, *P* < 0.05; **, *P* < 0.01; ***, *P* < 0.001; ****, *P* < 0.0001.

The stem cell distribution in the intestinal crypts was analysed via immunohistochemistry and immunofluorescence, which revealed that the number of stem cells increased with increasing piglet age (Figure 9A and B). Compared with that of the control piglets, the number of stem cells was significantly greater in the duodenum of the PEDV-infected 2-week-old and 3-week-old piglets (*P* < 0.05), significantly lower in the jejunum of the PEDV-infected newborn piglets (*P* < 0.0001), significantly greater in the jejunum of the PEDV-infected 3-week-old and 4-week-old piglets (*P* < 0.05), and significantly lower in the ileum of the PEDV-infected newborn piglets (*P* < 0.0001), but significantly greater in the PEDV-infected 4-week-old and 8-week-old piglets (*P* < 0.0001) (Figure 9C).

RT‒qPCR analysis of the stem cell marker Lgr5 revealed significant differences between different intestinal tissues of PEDV-infected piglets of different ages. Compared with those in the control piglets, Lgr5 expression levels were significantly greater in the duodenum of PEDV-infected 1-week-old and 4-week-old piglets (*P* < 0.01), significantly greater in the jejunum of PEDV-infected 4-week-old piglets (*P* < 0.001), and significantly greater in the ileum of PEDV-infected 8-week-old piglets (*P* < 0.0001). No apparent significant differences between the other groups were observed (Figure 9D).

## Discussion

In piglets of different ages infected with PEDV, clinical symptoms revealed that neonatal piglets had the highest mortality rate, the most severe diarrhoea, and the highest viral load. Postmortem examination revealed that PEDV infection in piglets resulted in obvious intestinal distension, thinning of the intestinal walls, and the presence of a large amount of yellowish liquid in the intestinal lumen. With increasing age, the clinical symptoms, diarrhoea severity, viral load, and postmortem changes gradually become milder [1].

HE staining revealed significant age-dependent differences in intestinal villus length and morphology. Normally, the ratio of villi to crypts in newborn piglets can reach a maximum of 6, which gradually decreases with increasing age, indicating that villi are longest in newborn piglets and gradually shorten with age [11]. Moreover, the width of villi gradually increases as the crypt depth increases with age, thus increasing the surface area of the small intestine tissue and improving food conversion and absorption rates [12]. PEDV infection-induced morphological changes in the small intestine are negatively correlated with piglet age. With a villus-to-crypt ratio of 1, PEDV-infected newborn piglets and 1-week-old piglets presented the most severe villus damage, which was significantly different from that of normally developing newborn piglets and 1-week-old piglets. PEDV infection-induced intestinal morphological changes clearly result in intestinal dysfunction with severe diarrhoea and even death [13]. The continuous morphological changes within the intestinal tissues of piglets of different ages are likely due to variations in diet composition and feed changes at different stages. The primary factor driving these changes is meeting the necessary dietary needs at various developmental stages. Additionally, changes in villi and crypts increase the surface area of intestinal tissues, improving nutrient absorption efficiency [14]. In the intestinal epithelium, lymphocytes are located mainly in the intercellular spaces of intestinal epithelial cells, where they exhibit highly active characteristics. During the normal development of piglet intestines, lymphocytes migrate mainly at the base of the intestinal villi, where they play a crucial role in maintaining intestinal health by acting as the first line of defence against intestinal viral infections [15].

Our data revealed a positive correlation between the number of lymphocytes in the intestinal epithelium and piglet age, which is consistent with the findings of previous studies by Wiarda et al. [16]. The fact that newborn and suckling piglets have lower epithelial lymphocyte numbers in their intestinal mucosa might explain their greater susceptibility to PEDV infection [17]. In fact, intestinal PEDV infection activates intestinal epithelial lymphocytes, resulting in increased numbers of epithelial lymphocytes leading to increased lymphocyte migration and infiltration, as well as a gradual relocation of intestinal epithelial lymphocytes toward the intercellular spaces and increased numbers of epithelial lymphocytes. This cellular positioning and migration of increased numbers of lymphocytes in the intestinal mucosa is suggested to maintain intestinal mucosal function and protect the body from the invasion of pathogenic microorganisms [18].

The intestinal epithelium serves as a robust barrier, effectively regulating the absorption of nutrients, inorganic salts, and water while significantly restricting the invasion of pathogenic microorganisms into the intestinal lumen [19]. Tight junctions are cell adhesion devices that act as barriers or channels in the space between adjacent epithelial cells. Moreover, it is also an important part of the mechanical barrier in the intestinal barrier and can maintain the integrity and permeability of the intestinal mucosal barrier [20]. In this study, we detected the expression of tight junctions and adhesion junctions in different intestinal tissues of piglets infected with PEDV via RT‒qPCR. The results revealed significant differences in expression between the infected and control groups. PEDV infection can cause changes in tight junctions and adhesion junctions between different intestinal tissues, destroying tight junction structures. ZO-1, claudin, occludin and E-cadherin expression was significantly lower in the PEDV-infected group than in the control group, suggesting that PEDV infection causes intestinal barrier damage. Compared with that in the control group, the expression of occludin was significantly greater. According to the relevant literature, an increase in occludin contributes to the invasion of PEDV [21]. The expression of claudin-3 increased but then decreased gradually. Given the role of claudin-3 in the intestinal epithelium, we speculate that the observed increases might represent an initial cellular repair response to PEDV stimulation. The subsequent decreases might reflect the restoration of barrier integrity as the piglets aged. PEDV infection destroys normal intestinal epithelial structure but does not affect the intestinal barrier function of piglets [22]. E-cadherin, a key component of adherens junctions, is increased in some tissues and ages following PEDV infection. This increase might contribute to epithelial repair. PEDV infection of different intestinal tissues of piglets at different ages leads to tight junction dysfunction, which ultimately leads to enhanced permeability between intestinal epithelial cells, aggravates the invasion of PEDV, and affects intestinal function [23].

Porcine small intestinal epithelial cells are the most important digestive and absorptive cells in the body and play crucial roles in nutrient absorption and intestinal immune regulation. In addition to being vital for nutrient absorption, intestinal epithelial cells play a significant role in resisting intestinal infections and regulating immune functions by acting as a barrier against large molecules and pathogenic microorganisms [24]. As the primary target for infection, PEDV utilizes small intestinal epithelial cells for replication and proliferation, which results in cellular shedding and ultimately intestinal damage and severe clinical symptoms such as diarrhoea and dehydration. The results of the present study revealed that PEDV infection induced incomplete villus morphology together with evidence of intestinal epithelial cell shedding. Notably, the impact of PEDV infection on these cells varies with age, as evidenced by the increased severity of intestinal epithelial cell shedding in younger piglets. Considering that intestinal epithelial cells primarily function in digestion and absorption, the shedding of these cells disrupts the digestive function of piglets through the inhibition of nutrient absorption.

Goblet cells make up the single-layer epithelial structure of the intestine and account for 5~10% of the epithelial cells in the small intestinal mucosa. Goblet cells are distributed throughout the entire small intestine epithelium and are present mainly on the villi, where they play crucial roles in the intestinal mucosal barrier [25]. PAS staining revealed a wide distribution of goblet cells on the intestinal villi. The number of goblet cells positively correlated with piglet age. Goblet cells secrete mucins, which are essential components of the mucous layer on the surface of the intestinal mucosa. In fact, the maintenance of intestinal epithelium integrity and intestinal environment homeostasis strongly depends on functional goblet cells. Previous research has indicated that loss of goblet cells or dysfunction of cup cell secretion is closely associated with intestinal diseases [26]. PEDV infection led to decreased goblet cell numbers in piglet small intestinal tissues, resulting in impaired secretion and incomplete intestinal mucosa. These observations confirm the varying susceptibility of piglets of different ages to PEDV infection and affirm the positive correlation between intestinal tissue damage and the number of goblet cells [27].

Paneth cells, specialized intestinal epithelial cells that synthesize lysozyme and antimicrobial peptides, play crucial roles in innate immune defence and gut microbiota composition control [28]. The expression of lysozymes and antimicrobial peptides in Paneth cells is regulated by both intrinsic and extrinsic mechanisms. For example, in mice, *Salmonella enterica* infection of Paneth cells triggers endoplasmic reticulum stress through intrinsic signalling pathways, which leads to reduced lysozyme production [29]. On the other hand, Paneth cells receive extrinsic cues from lymphocytes, including type 3 innate lymphoid cells and Th17 cells [30]. Cytokines produced by these lymphocytes upregulate lysozyme production in Paneth cells. This immune response preserves intestinal homeostasis by assisting in the defence against microbial and viral invasion. The results revealed that Paneth cells are located primarily in the intestinal crypts of the small intestine, which is consistent with findings reported by Ayabe et al. Additionally, changes in Paneth cell number increase with increasing piglet age [31]. Furthermore, significant differences were observed in Paneth cell numbers in different intestinal tissues of piglets before and after PEDV infection. PEDV infection in piglets results in increased Paneth cell numbers, resulting in increased secretion of antimicrobial peptides and other defence substances, ultimately contributing to the formation of a robust and effective defence mechanism. These observations suggest that PEDV-induced shedding of intestinal epithelial cells and intestinal damage occur because the number of Paneth cells is insufficient. This prevents the formation of an effective mucosal defence and results in intestinal epithelial cell infection and the development of inflammation [32].

Proliferating cells enable the assessment of cell proliferation and intestinal renewal. The number of proliferating cells, which are concentrated mainly in the intestinal crypts, is positively correlated with piglet age [33, 34]. PEDV infection resulted in a significant reduction in the number of proliferating cells in the duodenum, jejunum, and ileum of newborn piglets because of PEDV-induced shedding of intestinal epithelial cells. Consequently, the proliferating cells located in the intestinal crypts cannot meet the demands of renewing intestinal epithelial cells, which results in impaired intestinal development in newborn piglets, loss of intestinal function, and high mortality [35]. With increasing age, the effect of PEDV-induced proliferating cell loss gradually attenuated, as exemplified by increased numbers of proliferating cells in the intestinal crypts of aging piglets following infection.

Enteroendocrine cells are a diverse group of secretory cells that originate from intestinal stem cells. Although these cells make up less than 1% of all gastrointestinal epithelial cells, they play a significant role in regulating gastrointestinal motility and secretion, nutrient metabolism, and maintaining the gastrointestinal microenvironment [36]. The results of the present study revealed that PEDV-infected enteroendocrine cells are widely distributed in intestinal villi and crypts, which is consistent with the indirect immunofluorescence results. PEDV infection-induced changes in the number of enteroendocrine cells in piglets are associated with alterations in the morphology of the small intestine, leading to the shedding of intestinal villi. This ultimately results in a reduction in the number of enteroendocrine cells, which is consistent with the immunohistochemistry results reported by Jung et al. [37].

Intestinal stem cells—progenitor cells that enable the continuous proliferation and differentiation of intestinal epithelial cells—play crucial roles in intestinal development, epithelial cell renewal, and morphological changes [38, 39]. The results of the current study revealed that intestinal stem cells in piglets are located primarily in intestinal crypts. PEDV infection results in decreased intestinal stem cell numbers in newborn piglets, thus hindering the renewal of the intestinal epithelium in different intestinal tissues and inhibiting the digestive and absorptive functions of intestinal epithelial cells [40]. These observations explain why PEDV infection in newborn piglets results in intestinal wall thinning and transparency. As piglets age, the number of intestinal stem cells gradually increases, and their intestinal function gradually improves. Moreover, their intestinal epithelial cells cooperate with each other, enabling them to better resist viral invasion. This leads to the production of more rapidly proliferating TA (transit amplifying) cells, which differentiate into various types of intestinal epithelial cells, including absorptive cells, goblet cells, enteroendocrine cells, and Paneth cells, which complete the renewal of the intestinal epithelium [41]. The results of the present study demonstrated that older piglets have greater numbers of intestinal stem cells. Additionally, a comparison of intestinal stem cell numbers before and after PEDV infection revealed that older PEDV-infected piglets had a greater number of intestinal stem cells than their younger counterparts did. These findings indicate that while PEDV infection causes intestinal epithelial cell shedding, it also stimulates an increase in the number of intestinal stem cells, thus promoting their continuous differentiation to achieve the renewal of intestinal epithelial cells.

This study analysed changes in the intestinal morphology of piglets of different ages and revealed that the villus length was negatively correlated with crypt depth with increasing age. While the villus-to-crypt ratio decreases as piglets age, PEDV infection leads to a reduction in this ratio, which results in altered small intestine morphology. Moreover, the results of the current study revealed that PEDV infection in piglets of different ages leads to a decrease in the number of intestinal epithelial cells, which compromises the integrity of the intestinal epithelial barrier and results in diarrhoea. With increasing age, piglet intestinal tissue repair capacity improves, resulting in a more robust intestinal epithelial barrier and attenuating PEDV-induced damage to the small intestine. This observation explains why newborn piglets are more susceptible to PEDV. In conclusion, this study provides a theoretical foundation for the prevention of PEDV infection and research on the intestinal epithelial barrier.

## Data Availability

The data presented in this study are available upon request from the corresponding author.

## References

[1] Wang Q, Vlasova AN, Kenney SP, Saif L (2019) Emerging and re-emerging coronaviruses in pigs. Curr Opin Virol 34:39–49. 10.1016/j.coviro.2018.12.001 30654269 10.1016/j.coviro.2018.12.001 PMC7102852

[2] Lin F, Zhang H, Li L, Yang Y, Zou X, Chen J, Tang X (2022) PEDV: insights and advances into types, function, structure, and receptor recognition. Viruses 14:1744. 10.3390/v14081744 36016366 10.3390/v14081744 PMC9416423

[3] Jung K, Saif LJ, Wang Q (2020) Porcine epidemic diarrhea virus (PEDV): An update on etiology, transmission, pathogenesis, and prevention and control. Virus Res 286:198045. 10.1016/j.virusres.2020.198045 32502552 10.1016/j.virusres.2020.198045 PMC7266596

[4] Song D, Park B (2012) Porcine epidemic diarrhoea virus: a comprehensive review of molecular epidemiology, diagnosis, and vaccines. Virus Genes 44:167–175. 10.1007/s11262-012-0713-1 22270324 10.1007/s11262-012-0713-1 PMC7089188

[5] Wen Z, Xu Z, Zhou Q, Li W, Wu Y, Du Y, Chen X, Cao Y (2019) A heterologous “prime-boost” anti-PEDV immunization for pregnant sows protects neonatal piglets through lactogenic immunity against PEDV. Lett Appl Microbiol 69:258–263. 10.1111/lam.13197 31278766 10.1111/lam.13197 PMC7165963

[6] Jung K, Ha Y, Ha SK, Kim J, Choi C, Park HK, Kim SH, Chae C (2006) Identification of porcine circovirus type 2 in retrospective cases of pigs naturally infected with porcine epidemic diarrhoea virus. Vet J 171:166–168. 10.1016/j.tvjl.2004.09.002 16427593 10.1016/j.tvjl.2004.09.002 PMC7110590

[7] Shu LZ, Ding YD, Xue QM, Cai W, Deng H (2023) Direct and indirect effects of pathogenic bacteria on the integrity of intestinal barrier. Ther Adv Gastroenterol. 10.1177/17562848231176427 37274298 10.1177/17562848231176427 PMC10233627

[8] Skrzypek T, Valverde Piedra JL, Skrzypek H, Wolinski J, Kazimierczak W, Szymanczyk S, Pawlowska M, Zabielski R (2005) Light and scanning electron microscopy evaluation of the postnatal small intestinal mucosa development in pigs. J Physiol Pharmacol 56:71–87 16077196

[9] Choi J, Augenlicht LH (2024) Intestinal stem cells: guardians of homeostasis in health and aging amid environmental challenges. Exp Mol Med 56:495–500. 10.1038/s12276-024-01179-1 38424189 10.1038/s12276-024-01179-1 PMC10985084

[10] Wu H, Mu C, Xu L, Yu K, Shen L, Zhu W (2024) Host-microbiota interaction in intestinal stem cell homeostasis. Gut microbes 16:2353399. 10.1080/19490976.2024.2353399 38757687 10.1080/19490976.2024.2353399 PMC11110705

[11] Verdile N, Mirmahmoudi R, Brevini TAL, Gandolfi F (2019) Evolution of pig intestinal stem cells from birth to weaning. Animal 13:2830–2839. 10.1017/S1751731119001319 31199215 10.1017/S1751731119001319

[12] Degroote J, Vergauwen H, Wang W, van Ginneken C, De Smet S, Michiels J (2020) Changes of the glutathione redox system during the weaning transition in piglets, in relation to small intestinal morphology and barrier function. J Anim Sci Biotechnol 11:45. 10.1186/s40104-020-00440-7 32337030 10.1186/s40104-020-00440-7 PMC7178753

[13] Wang XY, Zhao TQ, Xu DP, Zhang X, Ji CJ, Zhang DL (2019) The influence of porcine epidemic diarrhea virus on pig small intestine mucosal epithelial cell function. Arch Virol 164:83–90. 10.1007/s00705-018-4061-x 30284628 10.1007/s00705-018-4061-x PMC7087301

[14] Wang X, Yin L, Geng C, Zhang J, Li J, Huang P, Li Y, Wang Q, Yang H (2025) Impact of different feed intake levels on intestinal morphology and epithelial cell differentiation in piglets. J Anim Sci 103:skae262. 10.1093/jas/skae262 39238159 10.1093/jas/skae262 PMC11705090

[15] Ma H, Qiu Y, Yang H (2021) Intestinal intraepithelial lymphocytes: Maintainers of intestinal immune tolerance and regulators of intestinal immunity. J Leukoc Biol 109:339–347. 10.1002/JLB.3RU0220-111 32678936 10.1002/JLB.3RU0220-111 PMC7891415

[16] Wiarda JE, Trachsel JM, Bond ZF, Byrne K, Gabler N, Loving C (2020) Intraepithelial T cells diverge by intestinal location as pigs age. Front Immunol 11:1139. 10.3389/fimmu.2020.01139 32612605 10.3389/fimmu.2020.01139 PMC7308531

[17] Jung K, Annamalai T, Lu Z, Saif L (2015) Comparative pathogenesis of US porcine epidemic diarrhea virus (PEDV) strain PC21A in conventional 9-day-old nursing piglets vs. 26-day-old weaned pigs. Vet Microbiol 178:31–40. 10.1016/j.vetmic.2015.04.022 25939885 10.1016/j.vetmic.2015.04.022 PMC7117181

[18] Kuka M, Iannacone M (2017) Intestinal flossing keeps pathogens at bay. Dev Cell 43:383–384 29161588 10.1016/j.devcel.2017.11.006

[19] Lin S, Mukherjee S, Li J, Hou W, Chao P, Liu J (2021) Mucosal immunity-mediated modulation of the gut microbiome by oral delivery of probiotics into Peyer’s patches. Sci Adv 7:eabf0677. 10.1016/j.devcel.2017.11.006 33980483 10.1126/sciadv.abf0677 PMC8115924

[20] Otani T, Furuse M (2020) Tight junction structure and function revisited. Trends Cell Biol 30:805–817. 10.1016/j.tcb.2020.10.001 32891490 10.1016/j.tcb.2020.08.004

[21] Li J, Li Y, Liu P, Wang X, Ma Y, Zhong Q, Yang Q (2022) Porcine epidemic diarrhea virus infection disrupts the nasal endothelial barrier to favor viral dissemination. J Virol 96:e0038022. 10.1128/jvi.00380-22 35435723 10.1128/jvi.00380-22 PMC9093128

[22] Luo L, Gu Z, Pu J, Chen D, Tian G, He J, Zheng P, Mao X, Yu B (2024) Synbiotics improve growth performance and nutrient digestibility, inhibit PEDV infection, and prevent intestinal barrier dysfunction by mediating innate antivirus immune response in weaned piglets. J Anim Sci 102:1023. 10.1093/jas/skae023 10.1093/jas/skae023 PMC10894507 38271094

[23] Landau D (2006) Epithelial paracellular proteins in health and disease. Curr Opin Nephrol Hypertens 15:425–429. 10.1097/01.mnh.0000232883.43093.76 16775457 10.1097/01.mnh.0000232883.43093.76

[24] Li Y, Wang J, Li Y, Wu H, Zhao S, Yu Q (2019) Protecting intestinal epithelial cells against deoxynivalenol and *E. coli* damage by recombinant porcine IL-22. Vet Microbiol 231:154–159. 10.1016/j.vetmic.2019.02.027 30955803 10.1016/j.vetmic.2019.02.027 PMC7172643

[25] Pelaseyed T, Bergström JH, Gustafsson JK, Ermund A, Birchenough G, Schutte A, van der Post S, Svensson F, Rodriguez-Pineiro A, Nystrom E, Wising C, Johansson M, Hansson G (2014) The mucus and mucins of the goblet cells and enterocytes provide the first defense line of the gastrointestinal tract and interact with the immune system. Immunol Rev 260:8–20. 10.1111/imr.12182 24942678 10.1111/imr.12182 PMC4281373

[26] Birchenough GM, Johansson ME, Gustafsson JK, Bergstrom JH, Hansson GC (2015) New developments in goblet cell mucus secretion and function. Mucosal Immunol 8:712–719. 10.1038/mi.2015.32 25872481 10.1038/mi.2015.32 PMC4631840

[27] Madson DM, Arruda PH, Magstadt DR, Burrough ER, Hoang H, Sun D, Bower LP, Bhandari M, Gauger PC, Stevenson GW, Wilberts BL, Wang C, Zhang J, Yoon KJ (2016) Characterization of porcine epidemic diarrhea virus isolate US/Iowa/18984/2013 infection in 1-day-old cesarean-derived colostrum-deprived piglets. Vet Pathol 53:44–52. 10.1177/0300985815591080 26113613 10.1177/0300985815591080

[28] Bevins CL, Salzman NH (2011) Paneth cells, antimicrobial peptides and maintenance of intestinal homeostasis. Nat Rev Microbiol 9:356–368. 10.1038/nrmicro2546 21423246 10.1038/nrmicro2546

[29] Bel S, Pendse M, Wang Y, Li Y, Ruhn K, Hassell B, Leal T, Winter S, Xavier R, Hooper L (2017) Paneth cells secrete lysozyme via secretory autophagy during bacterial infection of the intestine. Science 357:1047–1052. 10.1126/science.aal4677 28751470 10.1126/science.aal4677 PMC5702267

[30] Nakamura K, Sakuragi N, Takakuwa A, Ayabe T (2016) Paneth cell α-defensins and enteric microbiota in health and disease. Biosci Microbiota Food Health 35:57–67. 10.12938/bmfh.2015-019 27200259 10.12938/bmfh.2015-019 PMC4858879

[31] Ayabe T, Satchell DP, Wilson CL, Parks WC, SElsted ME, Ouellette AJ (2000) Secretion of microbicidal alpha-defensins by intestinal Paneth cells in response to bacteria. Nat Immunol 1:113–118. 10.1038/77783 11248802 10.1038/77783

[32] Nizet V, Ohtake T, Lauth X, Trowbridge J, Rudisill J, Dorschner RA, Pestonjamsp V, Piraino J, Huttner K, Gallo RL (2001) Innate antimicrobial peptide protects the skin from invasive bacterial infection. Nature 414:454–457. 10.1038/35106587 11719807 10.1038/35106587

[33] Goodlad RA (2017) Quantification of epithelial cell proliferation, cell dynamics, and cell kinetics in vivo. WIREs Dev Biol 6:e274. 10.1002/wdev.274 10.1002/wdev.274 28474479

[34] Xiao H, Wu MM, Tan BE, Yin YL, Li TJ, Xiao DF, Li L (2013) Effects of composite antimicrobial peptides in weanling piglets challenged with deoxynivalenol: I. Growth performance, immune function, and antioxidation capacity. J Anim Sci 91:4772–4780. 10.2527/jas.2013-6426 23965387 10.2527/jas.2013-6426

[35] Xiong X, Tan B, Song M, Peng J, Kim K, Yin Y, Liu Y (2019) Nutritional intervention for the intestinal development and health of weaned pigs. Front Vet Sci 6:46. 10.3389/fvets.2019.00046 30847348 10.3389/fvets.2019.00046 PMC6393345

[36] Steinert RE, Feinle-Bisset C, Geary N, Beglinger C (2013) Digestive physiology of the pig symposium: secretion of gastrointestinal hormones and eating control. J Anim Sci 91:1963–1973. 10.2527/jas.2012-6022 23307852 10.2527/jas.2012-6022

[37] Jung K, Miyazaki A, Saif LJ (2018) Immunohistochemical detection of the vomiting-inducing monoamine neurotransmitter serotonin and enterochromaffin cells in the intestines of conventional or gnotobiotic (Gn) pigs infected with porcine epidemic diarrhea virus (PEDV) and serum cytokine responses of Gn pigs to acute PEDV infection. Res Vet Sci 119:99–108. 10.1016/j.rvsc.2018.06.009 29909130 10.1016/j.rvsc.2018.06.009 PMC7111759

[38] Stange DE (2013) Intestinal stem cells. Dig Dis 31:293–298. 10.1159/000355231 24246977 10.1159/000355231

[39] Pinto D, Clevers H (2005) Wnt control of stem cells and differentiation in the intestinal epithelium. Exp Cell Res 306:357–363. 10.1016/j.yexcr.2005.02.022 15925592 10.1016/j.yexcr.2005.02.022

[40] Zhang S, Zhang S, Hou Y, Huang Y, Cai J, Wang G, Cao Y, Chen Z, Fang X, Bao W (2023) Porcine deltacoronavirus infection disrupts the intestinal mucosal barrier and inhibits intestinal stem cell differentiation to goblet cells via the notch signaling pathway. J Virol 97:e00689-23. 10.1128/jvi.00689-23 37289083 10.1128/jvi.00689-23 PMC10308910

[41] Clevers H, Loh KM, Nusse R (2014) An integral program for tissue renewal and regeneration: Wnt signaling and stem cell control. Science 346:1248012. 10.1126/science.1248012 25278615 10.1126/science.1248012

